# Simultaneous Presentation of Definite Vestibular Migraine and Definite Ménière's Disease: Overlapping Syndrome of Two Diseases

**DOI:** 10.3389/fneur.2018.00749

**Published:** 2018-09-10

**Authors:** Toshihisa Murofushi, Masahito Tsubota, Kyoko Kitao, Eriko Yoshimura

**Affiliations:** ^1^Department of Otolaryngology, Teikyo University School of Medicine Mizonokuchi Hospital, Kawasaki, Japan; ^2^Yoshimura ENT Clinic, Fujisawa, Japan

**Keywords:** Ménière's disease, vestibular migraine, endolymphatic hydrops, saccule, channelopathy

## Abstract

**Objectives:** To review the clinical records of patients that exhibited the clinical features of both vestibular migraine (VM) and Ménière's disease (MD) during each episodic vertigo attack and to discuss the possible pathophysiology of such combination of symptoms.

**Subjects:** Ten patients that were selected according to criteria based on a combination of the diagnostic criteria for definite MD and VM (9 females and one male, age: 22–54 years) were enrolled. They were required to show features of both diseases in each vertigo attack.

**Methods:** The patients' medical histories and pure-tone audiometry, cervical vestibular evoked myogenic potential (cVEMP), video head-impulse test (vHIT), and caloric test results were examined. cVEMP was recorded using 500 and 1,000 Hz short tone bursts (125dBSPL, air-conducted), 500 Hz-1,000 Hz cVEMP slope, an index of endolymphatic hydrops in the saccule was calculated using normalized amplitudes of p13-n23. For performing vHIT, each subject was seated 1.5 m in front of a target and asked to keep watching it as their head was passively rotated by the examiner. Their eye movements were evaluated using video-oculography while their head movements were recorded using inertial sensors.

**Results:** The patients were predominantly female. On average, the onset of migrainous headaches occurred 9 years earlier than the onset of vertigo attacks. All of the patients but one had migraines with auras. Five of the 10 patients had a family history of vertigo attacks accompanied by both migrainous and auditory symptoms. The patients mainly displayed hearing loss at low frequencies. Nine patients exhibited 500–1,000 Hz cVEMP slope < −19.9, which was suggestive of endolymphatic hydrops. None of the patients who underwent vHIT showed abnormal canal function. One patient showed unilaterally decreased caloric responses.

**Conclusions:** These patients presented with simultaneous MD and VM signs/symptoms might be referred to “VM/MD overlapping syndrome (VM/MD-OS)” as a new clinical syndrome.

## Introduction

Recently, diagnostic criteria for vestibular migraine (VM) and Ménière's disease (MD) have been published by the Barany Society (1, 2). To be diagnosed with having VM, vertigo attacks associated with migrainous symptoms such as headache, photophobia, and so on are required, while to be diagnosed with having MD, vertigo attacks associated with auditory symptoms including hearing loss are required. Both conditions are common, and their diagnosis is relatively straightforward in typical cases. However, clinicians sometimes encounter patients whose episodes of vertigo include features of both diseases (3, 4). Such conditions cannot be categorized into either of these two entities.

It has been well-known that some patients could have both diseases. Radtke et al. reported that lifetime prevalence of migraine was higher in the MD group (56%) than controls (25%, *p* < 0.001) (5). They stated that migraine and MD might have a pathophysiologic link. In the study of bilateral MD, Frejo et al. reported that 12% of the 398 patients with bilateral MD was associated with migraine. At this situation, patients who showed definite symptoms of both diseases in each episodic vertigo attack are of special interest.

Herein, we present a series of patients fulfilling the diagnostic criteria of both VM and MD, who presented with clinical features of both VM and MD during each episodic vertigo attack, and discuss the hypothesis of both disease manifestations to result from a common pathophysiology in the sense of being a true overlap syndrome (VM/MD overlapping syndrome, VM/MD-OS) rather than merely coincidence or co-morbidity.

## Subjects and methods

### Subjects

Patients that fulfilled the following criteria were enrolled into this study. Subjects were selected from the patients registered in our vertigo clinics in 2014–2017. The criteria were developed by combining the criteria for definite MD (2) and definite VM (1).

1. Having experienced at least 5 episodes of moderate to severe vestibular symptoms lasting 20 min−12 h.
2. Currently suffering from or having a history of migraines with or without auras, according to the International Classification of Headache Disorders, 3rd edition beta version (ICHD3β) (6).
3. Experiencing both of one or more characteristics of migraines and one or more fluctuating aural symptoms (hearing, tinnitus, or fullness) in the affected ear in a single vestibular episode during at least 50% of vestibular episodes.
4. Exhibiting audiometrically documented low to medium-frequency sensorineural hearing loss in one ear, on at least one occasion before, during, or after an episode of vertigo.
5. The patient's findings are not better accounted for by vestibular diagnoses other than VM, MD, or overlapping of VM and MD.

### Methods

The clinical records of the selected patients were reviewed. Information regarding the patients' age and sex, the affected side, age at the onset of migrainous headaches, age at the onset of vestibular symptoms, and family history of overlapping of vertigo, auditory symptoms and migrainous symptoms were reviewed. The results of pure-tone audiometry, cervical vestibular evoked myogenic potential (cVEMP) testing, video head impulse (vHIT) testing, and caloric testing were also reviewed.

### cVEMP

The Neuropack system (Nihon Kohden, Japan) was used to record the cVEMP. Active electrodes were placed on the upper half of each sternocleidomastoid muscle (SCM), while reference electrodes were placed on the lateral end of the upper sternum. A ground electrode was placed on the nasion. During the recording, the subjects were asked to lie in the supine position and raise their heads to contract the SCM. Five hundred Hz and 1,000 Hz air-conducted short-tone bursts (STB) (rise/fall time = 1 ms, plateau time = 2 ms, 125 dB SPL) were used to induce cVEMP. The other recording methods were the same as in previous studies (7–11). Using the normalized amplitude of p13-n23, the asymmetry ratio (AR) (500 Hz STB) and the 500-1,000 Hz cVEMP slope (cVEMP slope) were calculated as follows:

AR = 100 × (CAu-CAa)/(CAu+CAa)

cVEMP slope = 100 × (CA500-CA1000)/(CA500+CA1000)

CAu(a): normalized amplitude (p13-n23) of the unaffected (affected) side

CA500(1000): normalized amplitude (p13-n23) in response to 500 Hz (1,000 Hz) STB

According to results in previous studies (7, 11), the limits of the normal range were set at 41.6 for the AR and−19.9 for the cVEMP slope. The AR is an index used to detect unilateral saccular dysfunction, while the cVEMP slope is used to diagnose saccular endolymphatic hydrops (EH) (7, 11). In other words, an AR of >41.6 is indicative of unilateral saccular dysfunction, while a cVEMP slope of < -19.9 is indicative of saccular EH.

### vHIT

Eye-See-Cam system (Interacoustics, Denmark) was used for vHIT. The patients were subjected to passive high-acceleration, low-amplitude head rotations in the planes of the lateral, right anterior-left posterior (RALP), and left anterior-right posterior (LARP) semicircular canals (12). Each subject was seated 1.5 m in front of a target and asked to keep watching it as their head was passively rotated by the examiner. Their eye movements were evaluated using video-oculography while their head movements were recorded using inertial sensors. At least 8 valid head impulses were recorded in each plane of each semicircular canal. The VOR gains during the vHIT (eye velocity/head velocity) were automatically measured using software that computed the slope of the regression between head and eye velocity (13). When a mean gain in vHIT of < 0.7 for the vertical canals or < 0.8 for the lateral canals was detected and catch-up (corrective) saccades were observed, the relevant canal was regarded to be functioning abnormally (14).

### Caloric test

Caloric test was performed by irrigation of cold water (20°C) to the external ear canal (11, 15). Canal paresis (CP)% was calculated using maximum slow phase eye velocity measured by electronystagmography. CP%>20 was regarded as significant unilateral weakness of responses.

Consent concerning the use of clinical records was obtained from each patient in advance. This study was approved by the ethics committee of Teikyo University (TR-14-087-2).

## Results

A total of 10 patients who fulfilled the criteria were enrolled into this study (Tables 1–3).

**Table 1:** Summary of the patients' medical histories.

**No**.	**Age**	**Side**	**Aura**	**Family history**	**Onset age of headaches**	**Onset age of vertigo**	**Order of symptoms**
1	50–54	L	Yes	Yes	48	54	Fullness->vertigo->headache
2	30–34	R	Yes	No	16	30	Headache->tinnitus->vertigo
3	45–49	L	Yes	Yes	25	49	Fullness->headache->vertigo
4	50–54	R	Yes	No	51	54	Fullness->vertigo->headache
5	30–34	L	Yes	Yes	23	31	Headache->tinnitus->vertigo
6	45–49	L	Yes	No^*^	43	48	Fullness->headache->vertigo
7	50–54	R	Yes	Yes	49	50	Headache->tinnitus->vertigo
8	20–24	L	No	Yes	10	20	Tinnitus->vertigo->headache
9	30–34	L	Yes	No^*^	14	33	Tinnitus->vertigo->headache
10	45–50	L	Yes	No	43	43	Tinnitus->headache->vertigo
Mean	42.0				32.2	41.2

* *The patient did not have a family history of overlapping VM and MD, but did have a family history of migraines*.

**Table 2:** Patients' hearing levels at the time of the worst 4-tone average.

**No**.	**250 Hz (dB)**	**1,000 Hz (dB)**	**4,000 Hz (dB)**
1	70	65	55
2	40	25	25
3	55	55	35
4	35	15	15
5	30	10	10
6	55	45	10
7	40	40	55
8	35	15	10
9	45	35	20
10	60	40	20
Mean	46.5	34.5	25.5

**Table 3:** Vestibular function tests.

**No**.	**cVEMP AR**	**500-1,000 Hz cVEMP slope**	**vHIT**	**CP% in caloric test**
1	**100%**	−**100%**	LAP WNL	Left 2%
2	BAR	−**100%**	L WNL	Left 13%
3	17.6%	−**45.8%**	L WNL	X
4	BAR	NI	X	**Right 44%**
5	19.1%	−**27.8%**	X	Left 5%
6	**100%**	−**100%**	LAP WNL	0%
7	29.4%	−**21.5%**	LAP WNL	X
8	BAR	−**100%**	LAP WNL	Right 6%
9	−26.3%	−**31.5%**	LAP WNL	X
10	25.3%	−**29.20%**	LAP WNL	X

*Bolds indicate abnormal findings*.

*LAP WNL, normal in all canals*.

*L WNL, normal in the lateral canals*.

*BAR, bilateral absence of responses*.

NI, no information (absence of responses to both 500 Hz and 1000 Hz STB.)

*CP%, canal paresis %*.

*X, not done*.

### Age and gender

The 10 patients included one male and 9 females. Their ages ranged from 22 to 54 years (mean: 42.0 years).

### Affected side

The right ear was affected in 3 patients, while the left ear was affected in 7 patients.

### Aura

Nine of the 10 patients experienced visual auras.

### Family history

Five of the 10 patients had a family history of a similar combination of symptoms; i.e., vertigo associated with both of auditory and migrainous symptoms. Two patients had a family history of migraines, but no family history of associated vertigo. Three patients did not have a family history of migraines or vertigo associated with auditory symptoms.

### Onset of headaches and vertigo

The patients' migrainous headaches began at the age of 10–51 years (mean: 32.2), while their vertigo attacks began at 20–54 years (mean: 41.2). Thus, on average the migrainous headaches began 9.0 years earlier than the vertigo attacks.

### Order of symptoms during attacks

Four patients suffered auditory symptoms first, followed by vertigo and then migrainous headaches. Three patients developed migrainous headaches first, followed by auditory symptoms and then vertigo. The remaining three patients experienced auditory symptoms first, followed by migrainous headaches and then vertigo.

### Hearing loss

For each patient, hearing loss was assessed based on the hearing levels recorded at the time at which they exhibited their worst 4-tone average (AAO-HNS 1995) (16). Marked hearing loss was seen at low frequencies, whereas only mild hearing loss was detected at high frequencies. The subjects' mean hearing levels at 250, 1,000, and 4,000 Hz were 46.5, 34.5, and 25.5 dB HL, respectively (Table 2).

### cVEMP

No cVEMP were induced in response to air-conducted 500 Hz STB on the affected side in two patients. Responses to 500 Hz STB were bilaterally absent in three patients, and five patients had normal AR.

**Figure 1:**
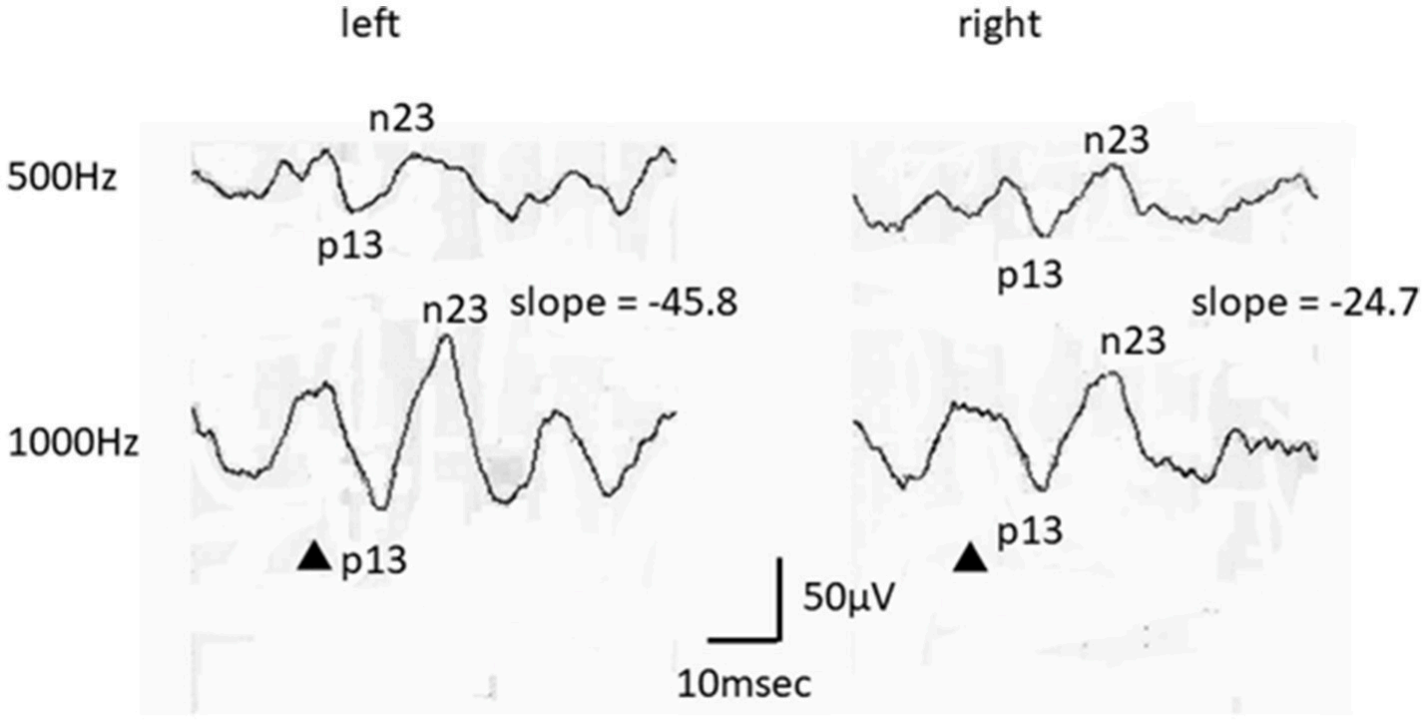
An example of the cVEMP findings obtained in this study (patient # 3, a 45–49-year-old female, left side affected). While this patient exhibited a normal AR, her cVEMP slope was suggestive of EH.

Concerning the patients' cVEMP tuning properties, nine patients were EH-positive on the affected side (cVEMP slope: < −19.9) (Figure 1), while one (patient #4) did not show any response to either 500 Hz or 1,000 Hz STB.

### vHIT

**Figure 2:**
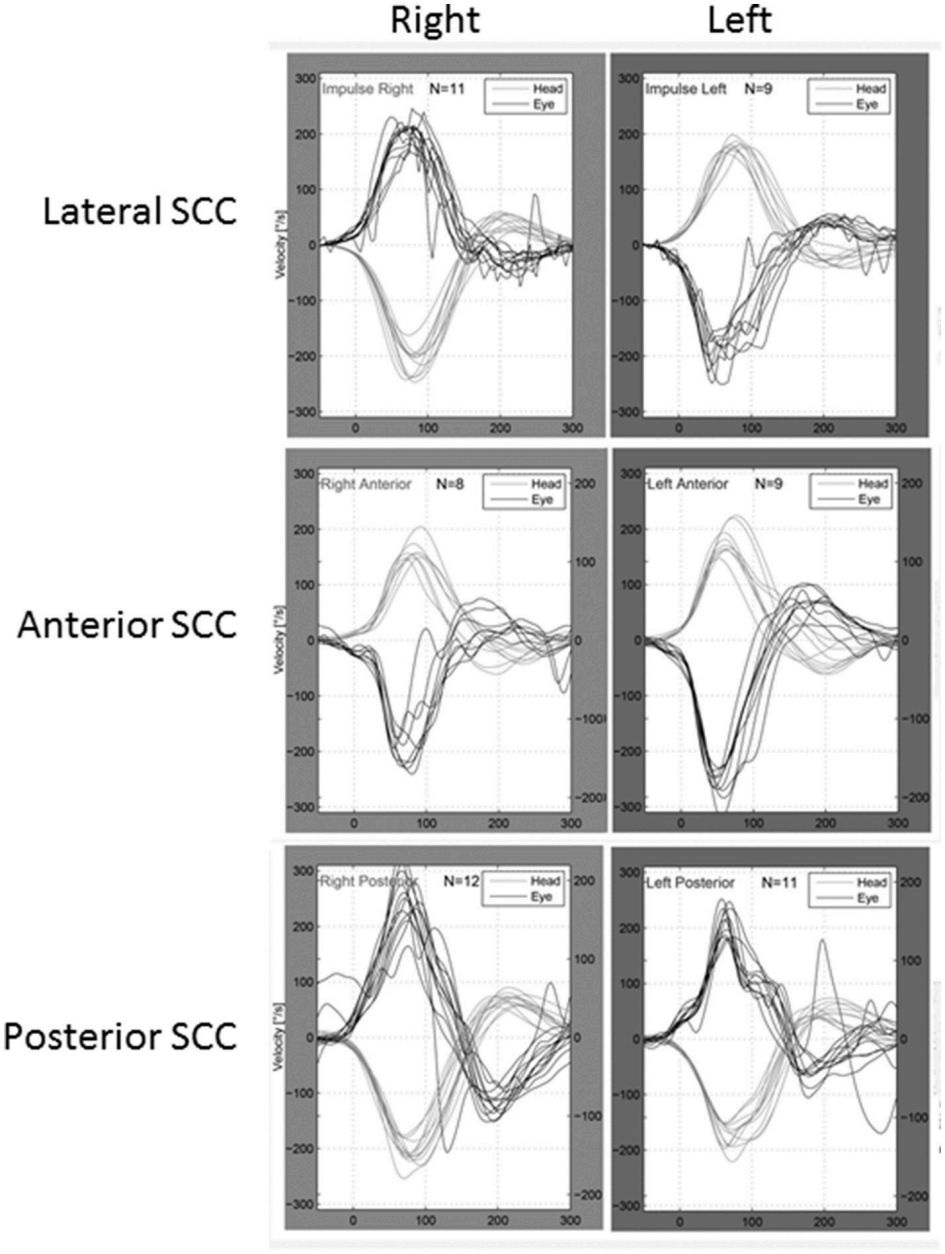
An example of the vHIT findings obtained in this study (patient # 9, a 30–34-year-old female, left side affected). She exhibited normal vestibulo-ocular reflexes in all canals. Thick lines: eye velocity, thin lines: head velocity.

Six patients underwent vHIT of all semicircular canals, and two patients only underwent vHIT of the lateral semicircular canals. One patient (#8) exhibited decreased gains in the anterior semicircular canal on the affected side (0.65) and the posterior semicircular canal on the contralateral side (0.56). One patient (#7) displayed a decreased gain in the posterior semicircular canal on the contralateral side (0.66). However, neither of them demonstrated catch-up saccades. Therefore, their vHIT results were not regarded as definitely abnormal. None of the other patients exhibited decreased gains during the vHIT (Figure 2).

### Caloric test

Six patients underwent caloric tests. Only one patient (#4) showed significant decrease of responses on the affected side (CP% = 44%).

## Discussion

In this study, we reported patients who were repeatedly presented with features of both MD and VM during each single vestibular episode in their recurrent vestibular episodes. Because half of the 10 patients reported they had family history of similar combination of symptoms, association of genetic factors was suggested. Because they had hearing loss at low frequencies and shifts of tuning property in cVEMP testing (9), they were considered to have EH. Such conditions might be best described as “VM/MD overlapping syndrome (VM/MD-OS).”

Lopez-Escamez et al. performed a large study examining the symptoms of patients with MD, VM, or probable VM. While most patients with MD had auditory symptoms and most patients with VM or probable VM had migraine symptoms, some patients exhibited overlapping symptoms of both conditions (3). However, their study excluded patients who fulfilled the diagnostic criteria for both VM and MD. On the contrary, our case series focused on patients who fulfilled the diagnostic criteria for both VM and MD at the time they experienced vertigo attacks.

Gurkov et al. reported the MRI findings of patients with VM and auditory symptoms (4). In their study, five patients fulfilled the diagnostic criteria for both definite VM and definite MD. Two of the five patients exhibited EH on MRI. Four of the five patients underwent electrocochleography, and all four of them had findings that were suggestive of EH.

Half of our patients had family history of simultaneous presentation of migrainous symptoms and fluctuating auditory symptoms including hearing loss during vestibular episodes. These patients might be regarded as having familial MD. Concerning familial MD, some reports have been published from European countries and Korea (17–22). Martin-Sierra et al. (21) reported that they identified 2 novel and rare heterozygous variants in the SEMA3D and DPT genes. However, none of patients in these families had migraine. Martin-Sierra et al. (22) also reported that they identified a novel missense variant in the PRKCB gene. Although one of the two patients had migraine, relationship between migraine and vertigo was not mentioned.

Frejo et al. reported that 12% of the 398 patients with bilateral MD showed association with migraine (17). Furthermore, Frejo et al. classified unilateral MD patients with cluster analysis (18). In their classification, 13% of the 988 MD patients were regarded as familial MD. Although half of our patients might be also classified as familial MD, our data were not conclusive. Even though our patients had aspects of familial MD, they were very rare cases because they had features of both MD and VM during each single vestibular episode in their recurrent vestibular episodes.

In the present study, patients who experienced episodic vertigo attacks and simultaneously showed signs/symptoms of both VM and MD in each attack exhibited the following features: (1) The patients were predominantly female; (2) The onset of migrainous headaches occurred earlier than the onset of vertigo attacks; (3) All of the patients but one had migraines with auras; (4) Many patients had a family history of vertigo attacks accompanied by both migrainous and auditory symptoms; (5) The patients exhibited hearing loss at low frequencies; (6) The cVEMP tuning property test suggested that most patients had EH.

Among these features, 1–4 suggested that the patients had VM spectrum disorders, while 5 and 6 suggested that they had MD spectrum disorders. It could be stated that these patients had VM accompanied by EH in the inner ear. However, it would be very hard to diagnose them as having either VM or MD. It would not describe their conditions accurately even if we regarded them as simply having both of VM and MD. Thus, their conditions might be best described as VM/MD-OS.

Regarding the pathophysiology of VM/MD-OS, neurogenic inflammation caused by migraine episodes might affect inner ear function because the trigeminal nerve innervates the blood vessels in the inner ear, and transient receptor potential cation channel subfamily V member 1 (TRPV-1), a nociceptor, was detected in the human endolymphatic sac (23, 24). Repetitive neurogenic inflammation in the inner ear might lead to secondary EH. This hypothesis could explain why the onset of vertigo episodes occurred later than the onset of migraine headaches in our patients. However, if this is the main pathophysiological mechanism underlying VM/MD-OS, then VM/MD-OS would be encountered more frequently, and vertigo attacks would occur more independently of migrainous headaches.

As an alternative hypothesis, it might be possible that genetic ion channel disorders could play a role in VM/MD-OS. It was one of interesting features of patients in this study that most patients (9/10) had migraine with aura. This prevalence is much higher than prevalence in patients with migraine in general (25) and in patients with vestibular migraine in general (3). The epidemiological study by Russel et al. (26) showed stronger association of genetic factors with migraine with aura than migraine without aura. Their finding suggested that VM/MD-OS might also have a strong genetic factor. The high frequencies of migraines with auras and a family history of vertigo attacks accompanied by both migrainous and auditory symptoms in the present study support this hypothesis. It has been reported that familial hemiplegic migraine type-1 is caused by a point mutation in the CACNL1A4 gene, which encodes the α_1_-subunit of voltage-sensitive P/Q-type calcium channels (27). A mutation affecting the same gene causes episodic ataxia type 2 (EA-2), in which patients often complain of migraines. Episodic ataxia type 3 (EA-3), a very rare type of EA, is characterized by migraines, vertigo, and tinnitus, although the gene associated with EA-3 has not been identified (28). Thus, VM/MD-OS might be a kind of channelopathy, like EA, which involves the inner ear as well as the brain.

As for other types of channel disorders that might cause VM/MD-OS, conditions that affect aquaporins, which function as water channels, should be considered (29). Among the various isoforms of aquaporin, disorders affecting aquaporin 4 (AQP4) might be associated with both migraines and inner ear diseases. Mhatre et al. detected anti-AQP4-immunoreactivity in the supporting cells in the cochlea, otolith organ, and semicircular canals in mice and rats (30). They also demonstrated that AQP4-knockout mice had impaired hearing. The dysregulation, including the hyperfunctioning, of AQP4 might result in dysregulated fluid homeostasis in the inner ear. Using a mouse model, Enger et al. showed that in cortical spreading depression (CSD), which is an essential process in migraines, extracellular glutamate accumulation increases due to AQP4-dependent glutamate release from astrocytes (31). The latter study suggested that AQP4 hyperfunction might result in CSD. AQP4 dysfunction might be associated with migrainous episodes as well as vertigo accompanied by hearing loss and other auditory symptoms.

It has been suggested that AQP2 might be another MD-associated isoform of aquaporin (27). Takeda et al. proposed that MD might be caused by dysregulation of the vasopressin-aquaporin system in the inner ear (32). However, AQP2 expression has not been detected in the brain, and no association between AQP2 and migraines has been reported.

As limitation of this study, we have to raise some points. First of all, the size of the study was not large. And the information concerning family history was not enough to describe pedigrees. We need further larger-sized precise study. Secondly, while we performed cVEMP tuning property test as a test for EH detection, we did not do other types of ED detection test such as glycerol test, electrocochleography and MRI (4, 9), although results of cVEMP tuning property test were consistent very well with glycerol cVEMP test results (9). We should consider these tests for confirmation of EH in patients with VM/MD-OS in the next step.

In conclusion, patients that simultaneously present with migrainous symptoms and vertigo attacks accompanied by hearing loss and other auditory symptoms seem to have VM accompanied by inner ear disorders including EH. Such conditions might be best described as VM/MD-OS, a new clinical syndrome.

## Author contributions

All authors contributed extensively to the work presented in this paper. All authors collected data. TM wrote the manuscript. MT, KK, and EY reviewed and edited the manuscript.

### Conflict of interest statement

The authors declare that the research was conducted in the absence of any commercial or financial relationships that could be construed as a potential conflict of interest.
